# Co-adjuvanting DDA/TDB liposomes with a TLR7 agonist allows for IgG2a/c class-switching in the absence of Th1 cells

**DOI:** 10.1038/s41541-023-00781-0

**Published:** 2023-12-22

**Authors:** Julie Zimmermann, Simon D. van Haren, Joann Diray-Arce, Ignatius Ryan Adriawan, Katharina Wørzner, Ricki T. Krog, Safia Guleed, Tu Hu, Rasmus Mortensen, Jes Dietrich, Sara M. Ø. Solbak, Ofer Levy, Dennis Christensen, Gabriel K. Pedersen

**Affiliations:** 1 Center for Vaccine Research, Statens Serum Institut, Copenhagen, Denmark; 2 Precision Vaccines Program, Boston Children’s Hospital, Boston, MA 02115 USA; 3 Department of Pediatrics, Harvard Medical School, Boston, MA USA; 4 Department of Drug Design and Pharmacology, University of Copenhagen, Copenhagen, Denmark; 5 Broad Institute of MIT and Harvard, Cambridge, MA USA; 6 Department of Immunology and Microbiology, University of Copenhagen, Copenhagen, Denmark

**Keywords:** Adjuvants, Antibodies, Lymphocyte differentiation

## Abstract

Class-switching to IgG2a/c in mice is a hallmark response to intracellular pathogens. T cells can promote class-switching and the predominant pathway for induction of IgG2a/c antibody responses has been suggested to be via stimulation from Th1 cells. We previously formulated CAF®01 (cationic liposomes containing dimethyldioctadecylammonium bromide (DDA) and Trehalose-6,6-dibehenate (TDB)) with the lipidated TLR7/8 agonist 3M-052 (DDA/TDB/3M-052), which promoted robust Th1 immunity in newborn mice. When testing this adjuvant in adult mice using the recombinant *Chlamydia trachomatis* (C.t.) vaccine antigen CTH522, it similarly enhanced IgG2a/c responses compared to DDA/TDB, but surprisingly reduced the magnitude of the IFN-γ+Th1 response in a TLR7 agonist dose-dependent manner. Single-cell RNA-sequencing revealed that DDA/TDB/3M-052 liposomes initiated early transcription of class-switch regulating genes directly in pre-germinal center B cells. Mixed bone marrow chimeras further demonstrated that this adjuvant did not require Th1 cells for IgG2a/c switching, but rather facilitated TLR7-dependent T-bet programming directly in B cells. This study underlines that adjuvant-directed IgG2a/c class-switching in vivo can occur in the absence of T-cell help, via direct activation of TLR7 on B cells and positions DDA/TDB/3M-052 as a powerful adjuvant capable of eliciting type I-like immunity in B cells without strong induction of Th1 responses.

## Introduction

Vaccines inducing strong cell-mediated immune responses are desired for protecting against major diseases caused by intracellular pathogens^1^. A cell-mediated immune response is characterized by strong expansion of T cells of the Th1 lineage and the production of antibodies (Abs) class-switched to the IgG subclass IgG2a/c. IgG2a/c activates Fcγ receptors distinct from those of other IgG subclasses^2^ and thus has distinct roles in protection against both bacterial^3,4^ and viral diseases^5–7^.

Th1 responses can be directly measured by assaying IFN-γ secretion from re-stimulated CD4 T cells. IFN-γ stimulates IgG2a/c Ab responses and inhibits the production of IgG1 in mice^8–10^, and Th1-inducing conditions such as viral infections or Th1-promoting adjuvants elicit high IgG2a/c responses^11–13^. Thus, whilst in humans there is no clear association of Th1 responses with a particular antibody subclass, IgG2a/c levels or ratios of IgG2a/c to IgG1 are often used to indicate Th1 responses in mice^14–16^.

The transcription factor T-bet (*Tbx21*) is a master regulator of Th1 immunity^17^. Conventionally associated with CD4 T cells, T-bet can also be expressed by NK cells and CD8 T cells. A recent body of literature also describes T-bet expression in a subset of B cells in aged mice^18^, in certain autoimmune diseases^19^, and in viral infections in mice and humans^20^. In addition, certain immunostimulators such as TLR7 and TLR9 agonists have been described to induce upregulation of T-bet expression in B cells in vitro^5,21^ and the induction of T-bet expression in B cells is required for class-switching to IgG2a/c^5,22^. Whilst IgG2a/c responses can be promoted by Th1 cells, it is possible that also IFN-γ produced by other immune cells could facilitate class-switching to this subclass or that the switching could occur completely independent of IFN-γ^23^.

Previously, we formulated the Th1-inducing adjuvant CAF®01; cationic liposomes formed by dimethyldioctadecylammonium (DDA) lipids, stabilized with the Mincle receptor agonist trehalose dibehenate (TDB)^24^. Based on results indicating that the TLR7/8 agonist 3M-052 is also able to induce Th1 responses^25,26^ we formulated a combination adjuvant, hypothesizing that 3M-052 could act in synergy with DDA/TDB to increase Th1 responses further and thus find use against diseases requiring strong cell-mediated immunity for protection. This adjuvant (named CAF®08b) effectively elicited Th1 responses in neonatal mice and protected against respiratory syncytial virus (RSV)^27^. In adult mice, however, we found that DDA/TDB/3M-052, despite increasing IgG2a/c titers, reduced the magnitude of the IFN-γ-producing Th1 cell response compared to DDA/TDB alone. Mechanistically, DDA/TDB/3M-052 turned on a type I immune transcriptional program directed by T-bet expression in both murine and human B cells. In vivo studies in mice revealed that T-bet expression occurred at the pre-germinal center (GC) stage and led to IgG2c class-switching without help from Th1 cells. Thus, IgG2a/c class-switching can occur both in a Th1-dependent and -independent manner and high levels of vaccine-induced IgG2a/c antibodies are not necessarily indicative of a high-magnitude Th1 response in vivo.

## Results

### Incorporating 3M-052 into DDA/TDB liposomes directs the antibody response toward the IgG2c subclass

**Fig. 1:**
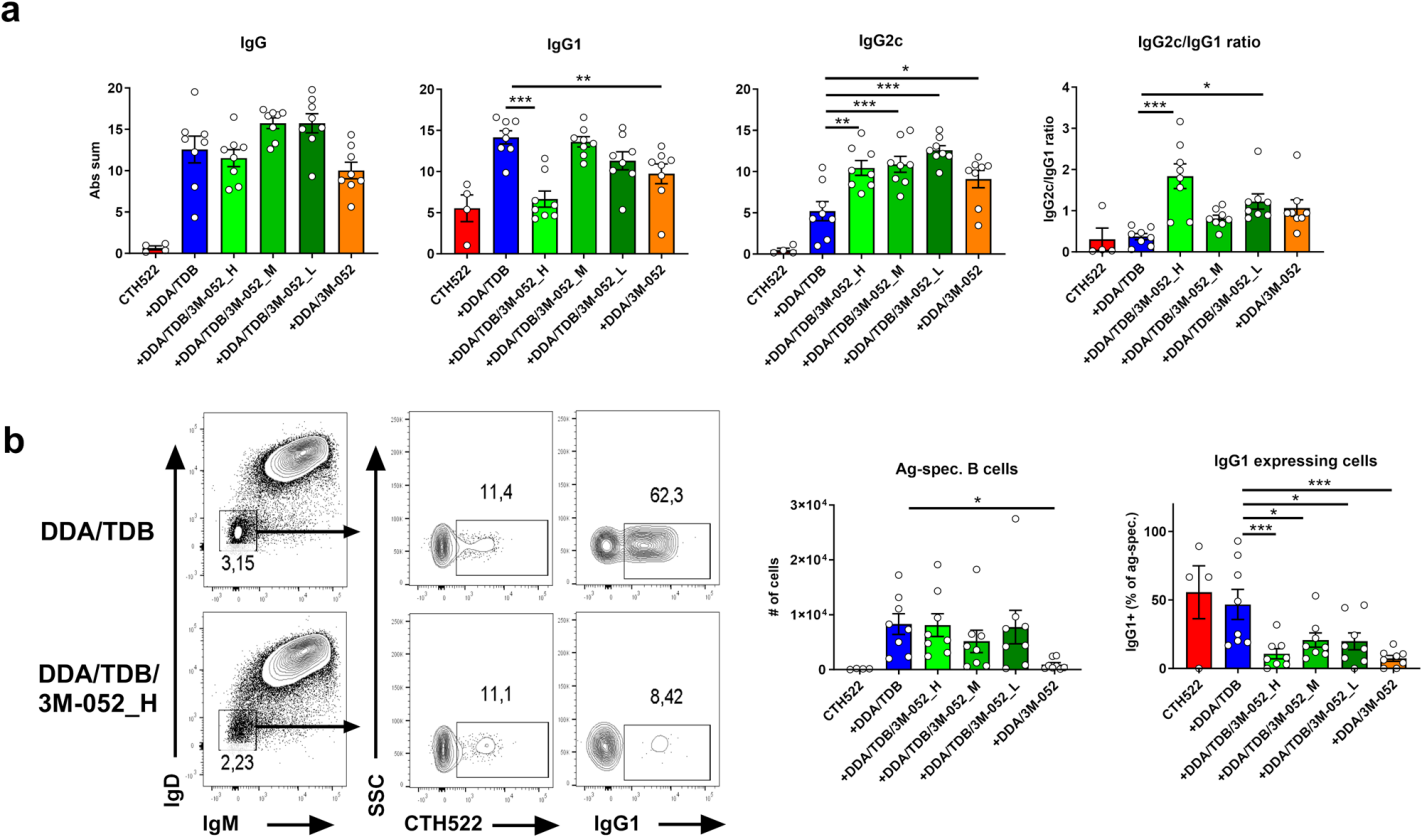
Liposomes containing a TLR7 agonist increase CTH522-induced IgG2c antibody responses. Mice were vaccinated subcutaneously with 2 µg of the recombinant protein antigen CTH522 either alone in the presence of the indicated cationic liposomal adjuvant. Mice were immunized twice, 3 weeks apart, and sacrificed 3 weeks after the second immunization. **a** Serum antigen-specific total IgG, IgG1, and IgG2c responses, displayed as a sum of absorbances (Abs sum). **b** IgG1 expression on antigen-specific B cells from draining lymph nodes was measured by gating on activated class-switched B cells (B220+IgD-IgM-) binding CTH522 coupled to a fluorophore. Adjuvants tested were cationic DDA liposomes containing either TDB (DDA/TDB), 3M-052 (DDA/3M-052), or both (DDA/TDB/3M-052). DDA/TDB/3M-052 was tested with three different doses of 3M-052 (H, M, L -corresponding to a dose of 10, 2, or 0.4 µg, respectively). The experiment was performed once. Each point represents individual mice (bars indicate mean + SEM). Statistically significant differences between groups are indicated by *, **, and *** (one-way ANOVA with Tukey´s correction for multiple group comparison, using the DDA/TDB group as reference and significance levels of *p* < 0.05, *p* < 0.01, and *p* < 0.001, respectively).

We sought to develop a strong Th1-promoting vaccine by co-adjuvanting the Th1/Th17-inducing adjuvant CAF®01 (DDA/TDB) with the TLR7/8 agonist 3M-052^25,26^. 3M-052 was formulated in DDA/TDB by the lipid film rehydration method, leading to highly stable cationic liposomes with size, polydispersity index (PDI), and zeta potential comparable to CAF01^27^. This combination adjuvant, CAF®08b (DDA/TDB/3M-052), was tested with a lead vaccine antigen candidate, CTH522^28^, targeting *Chlamydia trachomatis* for which Th1 responses are important for protection^29^. Initially, as an indicator for Th1 responses, the ability to promote IgG2c class-switching was tested and compared to that obtained when formulating the antigen in DDA/TDB liposomes alone. There were no significant differences in the total antigen-specific IgG responses, as indicated by the sum of absorbances, between the adjuvanted groups (Fig. 1a). DDA/TDB liposomes promoted a balanced IgG2c/IgG1 ratio (Fig. 1a). Additional incorporation of 3M-052 into the adjuvant (DDA/TDB/3M-052) skewed this ratio towards IgG2c (Fig. 1a). Particularly the DDA/TDB/3M-052_H formulation containing the highest amount of 3M-052 (10 µg/dose), gave significantly lower IgG1 responses than DDA/TDB (*p* < 0.001), whilst IgG2c titers were higher (*p* < 0.01). Incorporation of doses of 3M-052, 2 µg (DDA/TDB/3M-052_M), or 0.4 µg (DDA/TDB/3M-052_L), resulted in similar IgG1 responses to DDA/TDB, but higher IgG2c responses (*p* < 0.05). Similarly, DDA liposomes only containing 3M-052, but lacking the Mincle agonist TDB, elicited significantly lower IgG1 responses, but higher IgG2c responses, than DDA/TDB (*p* < 0.05) (Fig. 1a). We examined if incorporation of 3M-052 would direct class-switching away from IgG1 by analyzing antigen-specific B cells in the draining lymph nodes, using fluorophore-labeled CTH522 antigen and gating on activated class-switched (IgD-IgM-) B cells binding the probe. Similar numbers of antigen-specific B cells were found in the DDA/TDB/3M-052 and DDA/TDB groups, but in the latter on average 50% of antigen-specific B cells were IgG1 positive, while in all DDA/TDB/3M-052 groups, the IgG1 frequencies were significantly lower (*p* < 0.05) (Fig. 1b). Thus, incorporating the TLR7 agonist 3M-052 into DDA/TDB liposomes skewed IgG subclass antibody responses towards more IgG2c at the expense of IgG1, which is suggestive of a Th1 response.

### Incorporating 3M-052 into DDA/TDB liposomes abrogates Th1 responses

**Fig. 2:**
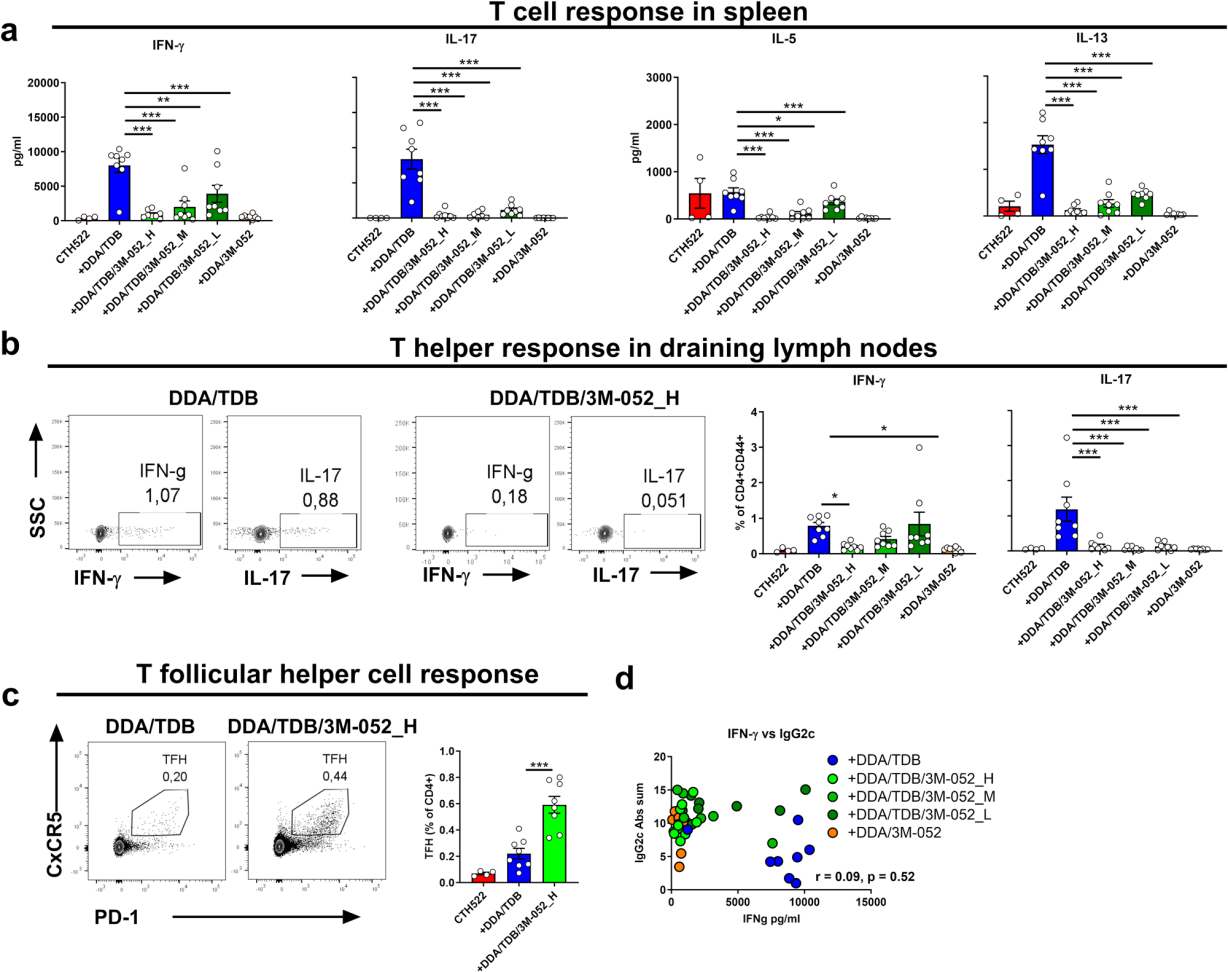
Adjuvanted CTH522-induced IgG2a/c responses do not correlate with Th1 responses. CB6F1 mice were vaccinated subcutaneously with 2 µg of the recombinant protein antigen CTH522 either alone in the presence of the indicated cationic liposomal adjuvant. Adjuvants tested were cationic DDA liposomes containing either TDB (DDA/TDB), 3M-052 (DDA/3M-052), or both (DDA/TDB/3M-052). DDA/TDB/3M-052 was tested with three different doses of 3M-052 (H, M, L -corresponding to a dose of 10, 2, or 0.4 µg, respectively). Mice were immunized twice, 3 weeks apart, and sacrificed 3 weeks after the second immunization. **a** T helper cell responses in the spleen were measured by re-stimulation with antigen and measuring secreted IFN-γ, IL-17, IL-5, and IL-13. **b** T-cell responses in draining lymph nodes were measured by intracellular staining of the indicated cytokines by flow cytometry. The cells were gated as CD4+CD44+ **c** T follicular helper (TFH) cell responses measured by flow cytometry by gating on CD4+B220-PD-1+CxCR5+ cells. **d** Lack of correlation between IFN-γ producing T cells (Th1) and IgG2c responses. Spearman correlation is calculated with two-tailed test. Groups consisted of 4 (antigen alone) or 8 (antigen + adjuvants) mice. The experiment was performed once. Each point represents individual mice (bars indicate mean + SEM). Statistically significant differences between groups are indicated by *, **, and *** (one-way ANOVA with Tukey’s correction for multiple group comparison, using the DDA/TDB group as reference and significance levels of *p* < 0.05, *p* < 0.01, and *p* < 0.001, respectively).

Since incorporating 3M-052 into DDA/TDB shifted the humoral immune response towards IgG2c, we anticipated a robust Th1 response by the combination adjuvant. However, contrary to our hypothesis, IFN-γ levels in supernatants from splenocytes re-stimulated with CTH522 were significantly lower in all groups containing the 3M-052 adjuvant than in the DDA/TDB adjuvanted group (*p* < 0.01) (Fig. 2a). Notably, the high dose of 3M-052 (DDA/TDB/3M-052_H) completely abrogated the systemic IFN-γ producing Th1 responses compared to DDA/TDB alone (Fig. 2a), despite providing the highest IgG2c/IgG1 ratio (Fig. 1a). Th17 responses were also completely abrogated when incorporating 3M-052 in DDA/TDB and Th2 responses, measured by IL-5 and IL-13 secretion, were significantly reduced (Fig. 2a). Similar reductions in splenic CD4 Th1 and Th17 cell responses were measured by intracellular cytokine staining (ICS) (Supplementary Fig. 1) and local Th1 responses in the draining lymph nodes (LNs), measured by ICS, were also significantly lower when DDA/TDB was combined with the high dose of 3M-052 adjuvant (DDA/TDB/3M-052_H; *p* < 0.05) (Fig. 2b). Similarly, frequencies of IL-17A producing T cells in draining LNs were lower in all groups receiving formulations containing 3M-052 compared to those obtained with DDA/TDB (*p* < 0.001). Since incorporating 3M-052 reduced Th responses of all major subsets, but provided similar or higher IgG2c antibody responses, we investigated the induction of T follicular helper cells (Tfh), using the DDA/TDB/3M-052_H formulation. Interestingly, despite reducing Th1/17 responses compared to DDA/TDB (Supplementary Fig. 2A), DDA/TDB/3M-052_H significantly increased Tfh responses (*p* < 0.001) (Fig. 2c) and, in line with effective Tfh cell help, the IgG2c response obtained by incorporating 3M-052 in DDA/TDB was long-lived and significantly higher than in the DDA/TDB alone group at 3 months post-immunization (*p* < 0.05) (Supplementary Fig. 2B). Overall, incorporation of the 3M-052 adjuvant in DDA/TDB liposomes elicited a strong IgG2c-skewed antibody response but abrogated Th1/2/17 responses in a 3M-052 dose-dependent manner. Thus, the levels of IFN-γ producing Th1 cells did not correlate with IgG2c responses (Fig. 2d).

### 3M-052 induces early transcriptional changes in the draining lymph nodes

**Fig. 3:**
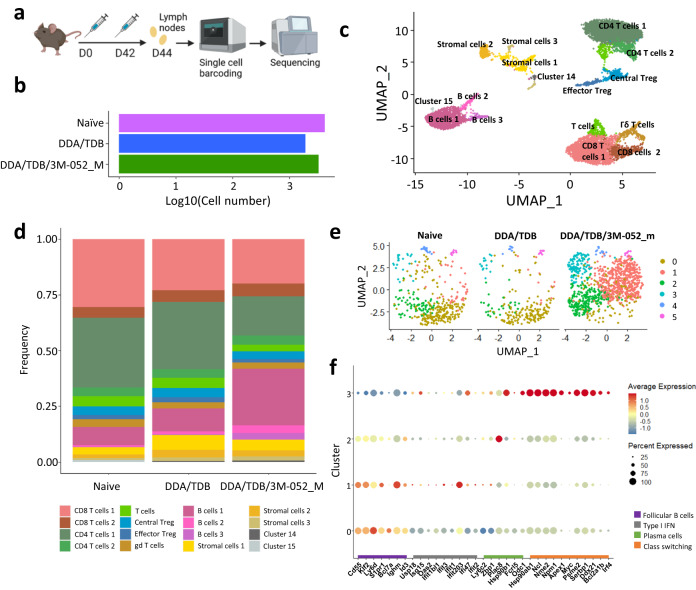
3M-052 adjuvantation of CTH522 induces early transcriptional changes in the draining lymph nodes. Mice were vaccinated s.c. with CTH522 in DDA/TDB or DDA/TDB/3M-052_M twice (given 6 weeks apart) and LNs were investigated 2 days after the last immunization. A naive control group was given tris-buffer. The cells from the LNs of three mice were pooled and single-cell RNA sequenced. **a** Schematic overview of the experiment, **b** Number of cells in each data file, **c** UMAP of integrated data file, **d** Frequency plot of the different cell populations from the different treatments, **e**, **f** The three B-cell clusters (B cells 1, B cells 2, and B cells 3) were re-clustered, **e** UMAP of the B-cell subsets, **f** Dotplot of a subset of the differentially expressed genes found when comparing cluster 1, 2, and 3 to cluster 0.

To investigate in more detail how the incorporation of 3M-052 influenced the induction of immune responses, we performed single-cell RNA-seq on cells from draining LNs in mice vaccinated with CTH522 in DDA/TDB or DDA/TDB/3M-052_M. To allow for broad profiling of immune signatures early after recall, we sampled LNs at 2 days after the last of two immunizations given 6 weeks apart (Fig. 3a). Low-quality cells were filtered out, and a transcriptome dataset with 9771 cells was obtained (Fig. 3b). To determine how the incorporation of 3M-052 in DDA/TDB influenced draining lymph node immune signatures, we performed unsupervised clustering and identified 16 clusters (Fig. 3c): three B-cell clusters (*Cd19, Ighd, Ighm*), two CD8+ T clusters (*Cd3g,Cd8a*), two CD4+ T cells clusters (*Cd3g,Cd4*), effector Tregs (*Foxp3, Ikzf2, Ctla4, Klrg1, Gzmb, Icos*), central Tregs (*Foxp3, Ikzf2, Ctla4, Sell, Bcl2*), γδ T cells (*Tcrg-C4, Tcrg-C2, Trdc*) and three Cd45-(*Ptprc*-) stromal cell populations. Separating the clusters into treatment types revealed that draining LNs from mice vaccinated with DDA/TDB/3M-052_M had a higher frequency of B cells compared to in the DDA/TDB group and naive mice (32% versus 12% and 9%, respectively) (Fig. 3d). In contrast, the frequencies of the T-cell populations were similar between the three groups.

Subclustering of the B-cell populations revealed 6 distinct clusters. B cells in naive mice and the DDA/TDB group were mainly found in cluster 0, whilst those in the DDA/TDB/3M-052_M group were found in three distinct clusters (1, 2, and 3) (Fig. 3e). Compared to cluster 0, cluster 1 was enriched for transcripts associated with early plasmablast formation (*Ly6c2 and Zbp1*)^30^. Notably, this cluster also contained several types of I IFN regulating genes, including *Ifit1, Ifit3, Usp18*, and *Isg15*, which are all activated downstream of TLR7^31^ (Fig. 3f). Compared to top cluster 0, cluster 2 had reduced expression of genes associated with follicular B cells (e.g., *Cd55, Klf2, Ighm*) and also had upregulated *Ly6c2*, whilst cluster 3 had strong upregulation of a number of genes associated with class-switching and GC responses (*Apex1, Myc, Nme2, Ncl, Npm1, Ddx21*). (Fig. 3f). Overall, these data demonstrated that incorporation of 3M-052 in DDA/TDB liposomes led to strong B-cell activation and expansion in the draining LNs. Furthermore, the upregulation of genes downstream of TLR7 in draining LN B cells suggested that 3M-052 delivered in DDA/TDB liposomes directly targets B cells in the draining LNs.

### Liposomes containing 3M-052 induce T-bet upregulation on pre-germinal center B cells

**Fig. 4:**
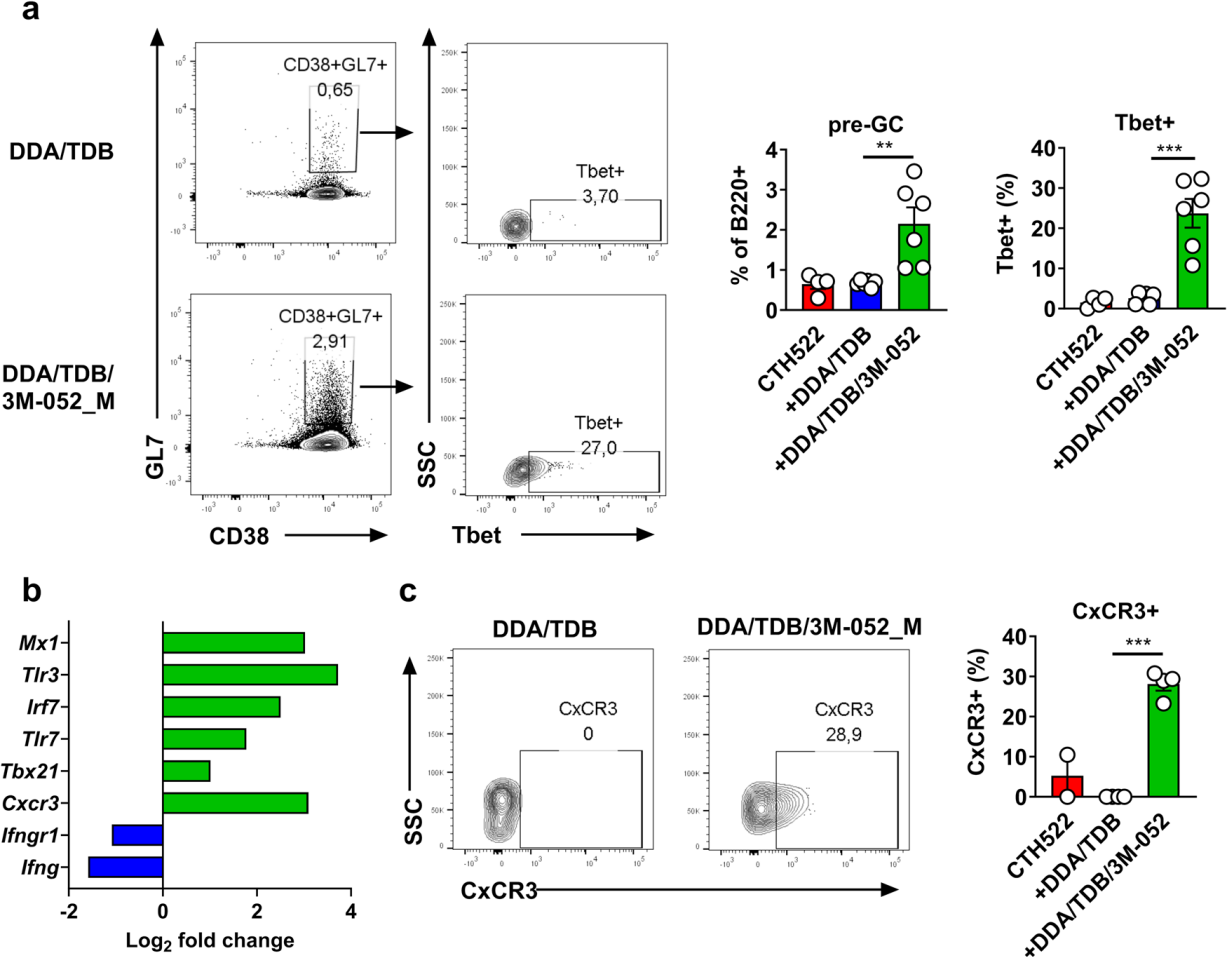
Cationic liposomes containing a TLR7 agonist induce Tbet expression in pre-germinal center B cells. Mice were injected subcutaneously with 2 µg of the recombinant protein antigen CTH522 either alone or in the presence of the indicated cationic liposomal adjuvant and draining lymph nodes collected 3 days later. **a** Representative staining of CD38+GL7+ pre-germinal center B cells (pre-GC) (pre-gated on B220+ cells) and their T-bet expression (left panels). Data are depicted as mean + SEM (right panels) and are representative of three independent experiments. **b** Differential gene expression of selected genes in isolated B cells from lymph nodes. Green bars display genes upregulated in the DDA/TDB/3M-052 group compared to the DDA/TDB alone group (pooled samples), while blue bars indicate downregulated genes. Isolated B cells were pooled from three mice per group and the experiment was performed once. **c** CxCR3 expression on pre-GC B cells. Left panels display representative staining and are pre-gated on B220+CD38+GL7+ cells. The right panel demonstrates mean + SEM (*n* = 3 mice per adjuvanted group) and the experiment was performed once. Statistically significant differences between groups are indicated by ** and *** (two-tailed unpaired *t*-test, with significance levels of *p* < 0.01 and *p* < 0.001, respectively.

TLR7 agonists can induce T-bet expression in B cells^5^. Inspired by the scRNA-seq data, which revealed early transcripts associated with GC responses and class-switching in LNs of mice immunized with DDA/TDB/3M-052_M, we next examined early T-bet expression 72 h after immunization. At this time point, we noticed a population of LN B cells displaying B220+CD38+GL7+, previously referred to as pre-GC B cells^32,33^, in mice immunized with CTH522 formulated in DDA/TDB/3M-052_m, whilst this population was found in lower frequencies in the DDA/TDB group (*p* < 0.01). Furthermore, within the DDA/TDB/3M-052_M these had high T-bet expression with on average 20 % being T-bet positive (significantly higher than the in the DDA/TDB group, *p* < 0.001) (Fig. 4a). qPCR on isolated B cells at 72 h post-immunization identified the gene encoding for T-bet (*Tbx21)* as being upregulated in mice having received DDA/TDB/3M-052_M compared to DDA/TDB. Other upregulated genes included those encoding for TLR7, Mx1, and IRF7, all involved in TLR7 signaling or TLR7-promoted antiviral immunity, while genes encoding for IFN-γ and IFN-γR1 were downregulated in the group having received DDA/TDB/3M-052_M compared to DDA/TDB (Fig. 4b). In line with previous studies describing that CxCR3 is upregulated in T-bet+ B cells^34,35^, qPCR and surface staining for CxCR3 also revealed high expression of this receptor in pre-GC B cells from DDA/TDB/3M-052 immunized mice (Fig. 4b, c). Thus, 3M-052 stimulated early B-cell activation and expression of T-bet prior to GC formation.

### In vitro stimulation of B cells with DDA/TDB/3M-052 liposomes induces T-bet upregulation and secretion of IgG2c

**Fig. 5:**
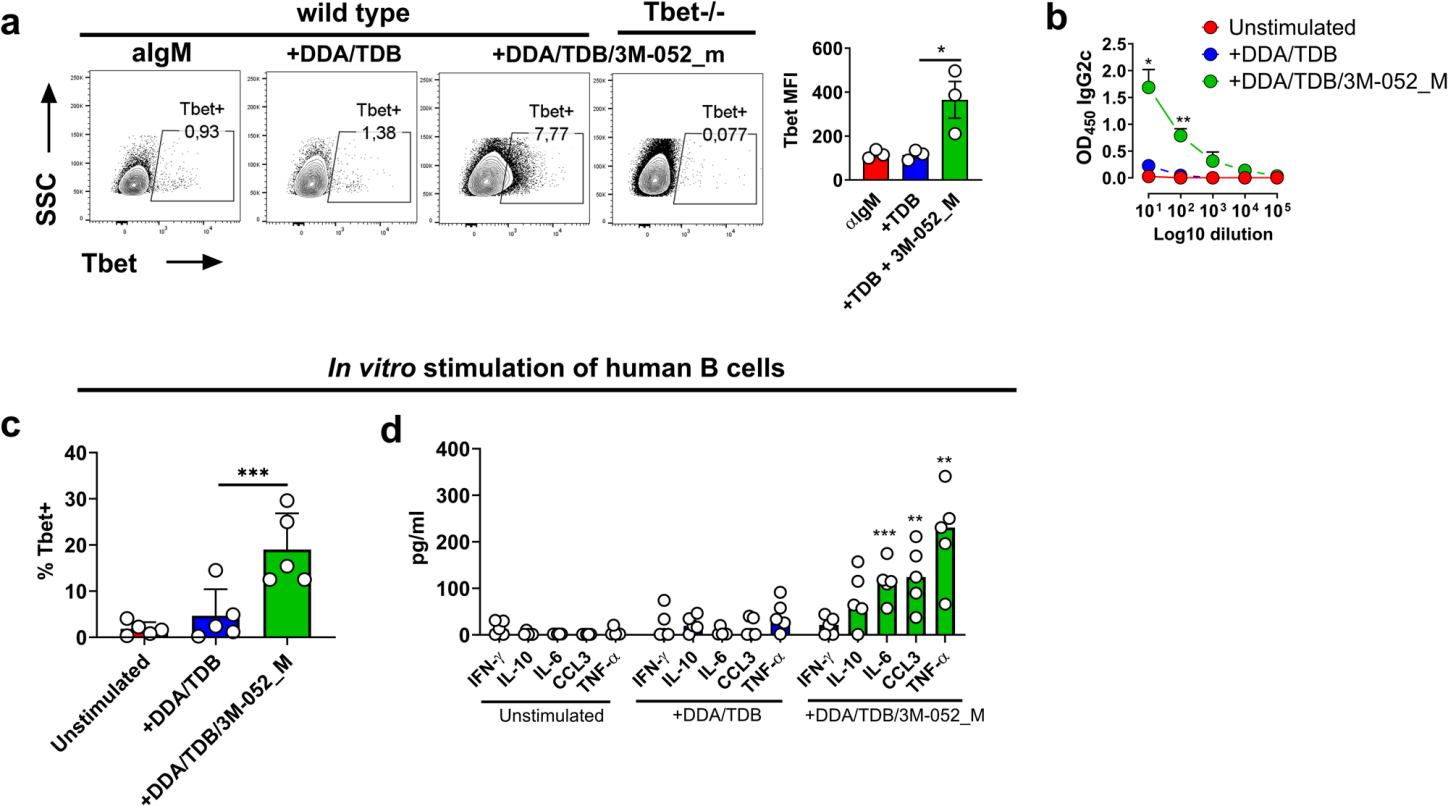
Cationic liposomes containing a TLR7 agonist promote Tbet expression and IgG2c secretion in vitro. **a** Wild type or T-bet-/- splenic isolated B cells were stimulated in vitro with anti-IgM in combination with cationic (DDA) liposomes containing TDB or TDB+3M-052 and evaluated for T-bet expression 24 h later. **b** IgG2c was measured in supernatants after 5 days of B-cell culture with either DDA/TDB or DDA/TDB/3M-052. **c** Naive human B cells (CD20+CD27–) were isolated and stimulated in vitro for 24 h before staining for T-bet. **d** IFN-γ, IL-10, IL-6, CCL3, and TNF-α were measured in supernatants from the in vitro cultures. Data are depicted as mean + SEM of three independent experiments. Statistically significant differences between the DDA/TDB and DDA/TDB/3M-052 groups are indicated by *, **, and *** (two-tailed unpaired *t*-test, with significance levels of *p* < 0.05, *p* < 0.01, and *p* < 0.001, respectively).

To investigate whether DDA/TDB/3M-052 liposomes could directly stimulate B cells to upregulate T-bet and undergo class-switching to IgG2c, we examined in vitro stimulation of isolated B cells. Indeed in vitro stimulation of isolated naive B cells with DDA/TDB/3M-052_M induced T-bet expression and secretion of IgG2c (Fig. 5a, b). In contrast, DDA/TDB did not induce T-bet expression nor IgG2c secretion from direct activation of murine B cells. The DDA/TDB/3M-052 liposomes also induced significantly higher T-bet expression in human naive B cells isolated from peripheral blood compared to DDA/TDB after 24 h of stimulation (Fig. 5c). We did not evaluate antibody secretion in the human cultures, as, there is no clear association of Th1 responses with a particular antibody subclass in humans. However, human B cells produced significantly increased levels of a range of cytokines, including IL-6 and TNF-α following 72 h stimulation with DDA/TDB/3M-052 compared to DDA/TDB (Fig. 5d). Thus, these findings indicated that DDA/TDB/3M-052 directly activated both murine and human B cells.

### B-cell-intrinsic TLR7 expression is required for 3M-052-liposome-induced IgG2c class-switching

**Fig. 6:**
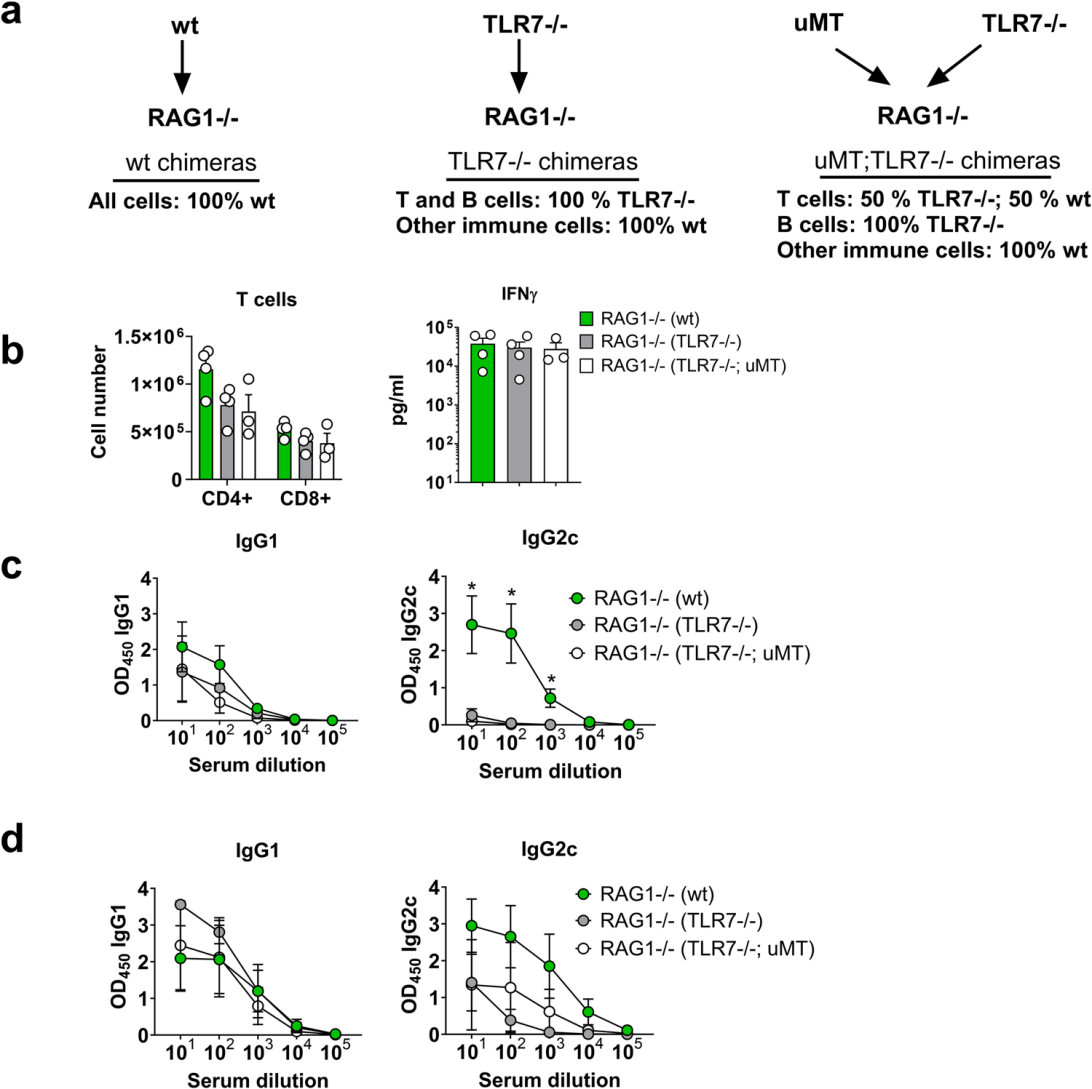
B-cell TLR7 expression is required for IgG2c switching in response to liposomes containing a TLR7 agonist. Bone marrow chimeras lacking B-cell-intrinsic TLR7 expression were generated by cell transfer to RAG1-/- mice. The mice were immunized with the CTH522 antigen formulated in cationic DDA liposomes containing TDB and 3M-052, using a 3M-052 dose of 2 µg (DDA/TDB/3M-052_M). The vaccine was given in two doses 3 weeks apart. **a** Overview of the different chimeras. **b** Number of T cells in the spleen (left panel) and Th1 responses, measured as IFN-γ measured in supernatants after re-stimulation with vaccine antigen (right panel). **c** Antigen-specific IgG1 and IgG2c responses measured in serum 1 week after the second immunization. **d** Antigen-specific IgG1 and IgG2c responses were measured at the end of the study (3 weeks after the second immunization). Groups consisted of 3–4 mice. The experiment was performed once. Data are depicted as mean + SEM. Statistically significant differences between groups are indicated by * (One-way ANOVA with Tukey´s correction for multiple group comparison, using the RAG1-/- (wt) group as reference and significance levels of *p < 0.05).

As DDA/TDB liposomes containing 3M-052 directly stimulated murine B cells to class-switch to IgG2c upon in vitro stimulation, we investigated if T-cell-help or B-cell intrinsic factors were the most important determinant for IgG2c class-switching in response to DDA/TDB/3M-052 in vivo. To generate mice in which only B cells lacked TLR7 expression, RAG2-/- mice received bone marrow which was either wt, TLR7-/- or a mixture of TLR7-/- and µMT (from B-cell-deficient mice). RAG2-/- mice that received TLR7-/- bone marrow thus lacked TLR7 on both T and B cells. Those mice that received mixed TLR7-/-;µMT, lacked TLR7 exclusively on B cells (as all B cells in these mice were from the TLR7-/- mice), whilst TLR7 expression on T cells was intact. In all chimeras, dendritic cells (DC) had intact TLR7, as dendritic cells are intact in both RAG1-/- mice and µMT mice (Fig. 6a). Thus, conventional DC-Th1 driven class-switching to IgG2c^36^ should thus be indifferent in all the chimeras. When immunized with CTH522 in DDA/TDB/3M-052_M, all chimeras indeed induced comparable Th1 responses, as measured by their ability to produce IFN-γ after antigen re-stimulation (Fig. 6b). All groups also mounted an IgG1 response, while mice that received wt bone marrow displayed higher IgG2c responses compared to those receiving TLR7-/- or TLR7-/-;µMT bone marrow at 1 week after the second immunization (Fig. 6c). There was also a tendency towards higher IgG2c responses in mice that had received wt bone marrow compared to TLR7-/- or TLR7-/-;µMT bone marrow at 3 weeks post the second immunization, although not statistically significant (*p* = 0.054 and 0.073, respectively). Thus, a Th1 response was insufficient to elicit optimal class-switching to IgG2c in response to DDA/TDB/3M-052_M, whereas TLR7 was required on B cells to drive preferential IgG2c switching in vivo.

### Th1 cells are not required to induce IgG2c class-switching in response to DDA/TDB/3M-052 in vivo

**Fig. 7:**
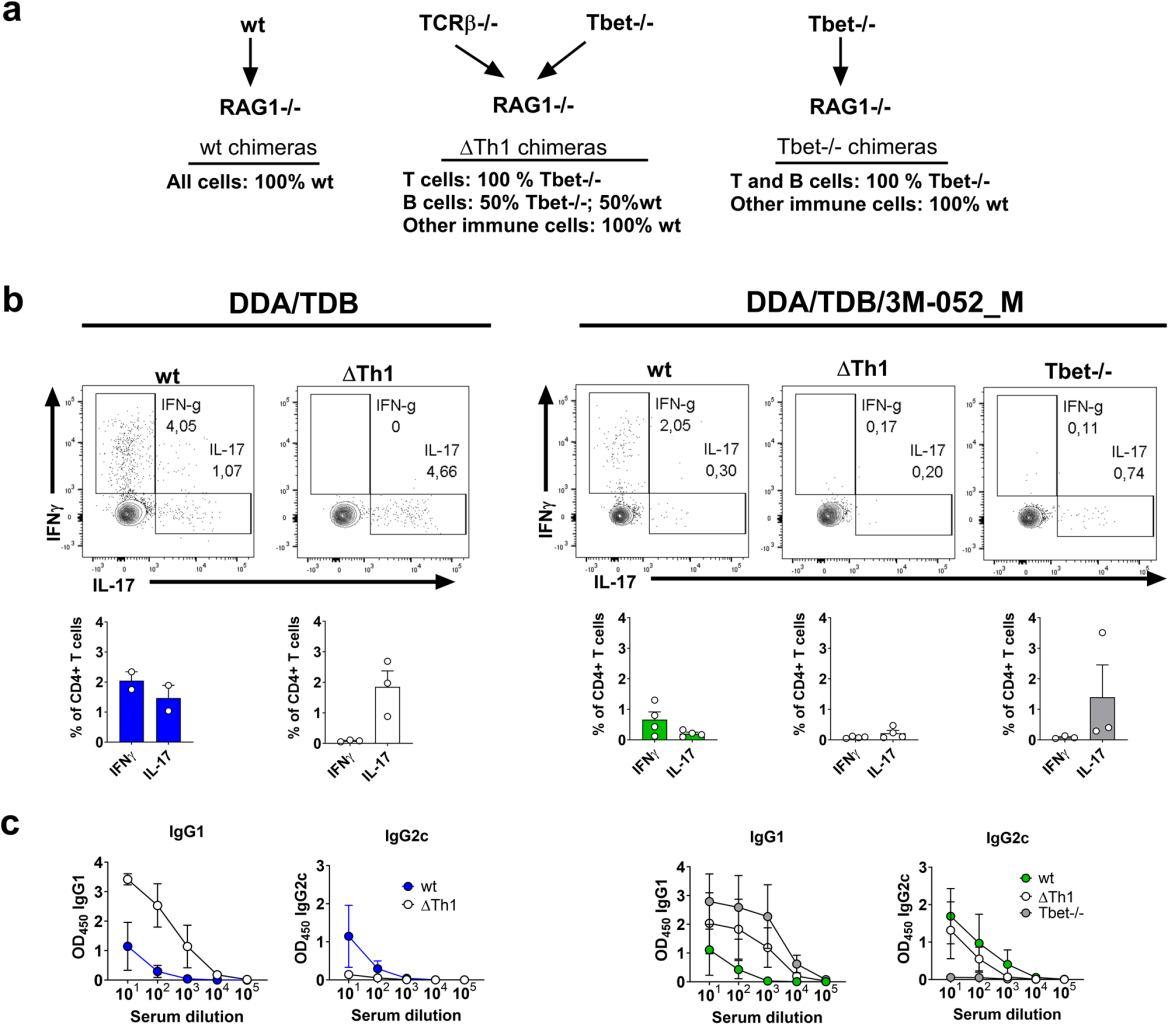
Th1 cells are redundant for IgG2c responses to TLR7-containing adjuvants. Bone marrow chimeras lacking Th1 cells, or lacking T-bet in both T and B cells, were generated by cell transfer to RAG1-/- mice. The mice were immunized with the CTH522 antigen formulated in cationic DDA liposomes containing either TDB (DDA/TDB) or TDB and 3M-052 (DDA/TDB/3M-052), using the low dose of 3M-052 (2 µg). The mice received three doses each 3 weeks apart. **a** Overview of the different chimeras. **b** Th1 and Th17 responses measured by intracellular cytokine staining on splenocytes after immunization with DDA/TDB (left panel) or DDA/TDB/3M-052 _M (right panel) after gating on CD4+CD44+ cells. **c** Antigen-specific IgG1 and IgG2c responses measured in serum 1 week after the second immunization with DDA/TDB (left panel) or DDA/TDB/3M-052_M (right panel). Groups consisted of 3–4 mice. The experiment was performed once. Data are depicted as mean + SEM.

The prevailing view is that Th1 cells are the main driver of IgG2c switching. As IgG2c switching in response to DDA/TDB/3M-052_M required direct activation of TLR7 in B cells and since IgG2c switching in vitro could take place in the absence of T cells, we hypothesized that DDA/TDB/3M-052_M-induced IgG2c class-switching could occur in the complete absence of Th1 cell help in vivo. To investigate this, we generated mixed bone marrow chimeras exclusively lacking Th1 cells (ΔTh1), but retaining the capacity to provide T-cell help, by transferring a mixture of TCRβ-/- and T-bet-/- cells into RAG2-/- mice, and evaluated IgG2c switching in response to antigen given in DDA/TDB or DDA/TDB/3M-052_M. We also made chimeras lacking T-bet in both B and T cells (T-bet-/-) (Fig. 7a). Mice were immunized with two doses of CTH522 antigen in combination with either DDA/TDB or DDA/TDB/3M-052_M adjuvant. All mice were successfully engrafted with B and T cells (Supplementary Fig. 3A, B) and, as expected, the ΔTh1 mice lacked IFN-γ-producing T cells, while their Th17 response was intact in response to DDA/TDB adjuvanted vaccine, as measured by ICS on splenocytes (Fig. 7b). The corresponding DDA/TDB/3M-052_M immunized group lacked both Th1 and Th17 cells (Fig. 7b), in line with the poor ability of DDA/TDB/3M-052_M to stimulate a Th17 response (Fig. 2). In the DDA/TDB groups, the ΔTh1 mice had a higher IgG1 response than the corresponding wt group, whilst IgG2c levels were highest in wt mice (Fig. 7c). In mice immunized with DDA/TDB/3M-052_M, both wt and ΔTh1 mice displayed IgG2c responses, while mice lacking T-bet in both B and T cells mounted no IgG2c response (Fig. 7c). When immunizing with a third vaccine dose, IgG2c responses were also seen in the DDA/TDB vaccinated ΔTh1-group and only the T-bet-/- group (immunized with DDA/TDB/3M-052_M) completely lacked IgG2c responses (Supplementary Fig. 3C, D). Overall, these data demonstrated that class-switching to IgG2c could occur in the complete absence of Th1 cells, but not when T-bet was lacking on both B and T cells.

## Discussion

In this study, we demonstrate that a liposomal adjuvant incorporating a TLR7 adjuvant can induce high-magnitude IgG2c antibody responses via direct activation of B cells and that these responses do not require efficient Th1 priming in vivo. Strategies to promote Th1 responses by vaccination are desired to protect against intracellular pathogens, such as Mycobacterium tuberculosis and Chlamydia trachomatis^1^. As switching to the IgG2a/c subclass in mice can be stimulated by a Th1-directed immune response^8,9,37^, direct measurements of Th1-derived IFN-γ are often corroborated by, or equated to, measuring IgG2a/c levels or IgG2a/c to IgG1 ratios as a surrogate for Th1 immunity^16,38,39^. The transcription factor T-bet was initially characterized in T cells for its role in direct Th1 differentiation^40^ and reduced switching to IgG2a/c in T-bet deficient mice was associated with insufficient Th1 help^41^. Later studies demonstrated that T-bet can also be stimulated in B cells and B-cell-specific T-bet ablation led to impaired IgG2a/c responses^22,42^. While Th1-derived IFN-γ may be the predominant path to T-bet expression and IgG2a/c switching, this can also be stimulated in the absence of IFN-γ signaling^23^. Thus, although T-bet is required for IgG2a/c class-switching, the exact mechanisms guiding this response are not completely resolved.

In search for Th1-inducing adjuvants, we combined a TDB-based cationic liposomal adjuvant (DDA/TDB; CAF®01) with the TLR7/8 agonist 3M-052, as both of these immunostimulators promote Th1 immunity^25,43^. We found that this adjuvant combination (CAF®08) effectively stimulated Th1 responses and IgG2c switching in newborn mice^27^. However, although DDA/TDB/3M-052 also induced high levels of IgG2c in adult mice, the Th1-derived IFN-γ responses were much lower than those observed when giving DDA/TDB liposomes alone, likely illustrating age-specific responses to combined TLR7 and Mincle stimulation^44^. One possibility for this difference between adult and neonatal mice could be that the strong Type I IFN response observed early after immunization in the adult mice was detrimental to the T-cell responses, whereas the type I IFN responses may be impaired in neonatal mice^45^. It should be noted that although 3M-052 is an agonist for both TLR7 and TLR8, mice do not express a functional TLR8 and thus the effects of this pathway could not be determined in the murine model.

The 3M-052 immunostimulator is a promising adjuvant capable of boosting Th1 responses when formulated in o/w emulsion^46^, liposomes^26^, or as a nanosuspension adsorbed to alhydrogel^25^. In these studies, the incorporation of 3M-052 in the different carriers increased both IgG2a/c responses and the levels of IFN-γ producing T cells. When incorporated in DDA/TDB liposomes, 3M-052 reduced Th1 responses at 3M-052 doses suggested as optimal^25^. However, although DDA/TDB/3M-052 was not effective at Th1 induction, this adjuvant is promising with respect to the strong B-cell activation and the potential of strongly activating B cells without induction of Th1/2/17 responses may be desirable for particular disease targets, such as HIV^47,48^. DDA/TDB/3M-052 also induced Tbet-directed elicitation of IgG2c responses, which can activate Ab-dependent cellular cytotoxicity (ADCC) and complement^49^ and control virus infections more effectively than other IgG isotypes^5–7^. T-bet+ B cells may also mediate antiviral immunity by other effector mechanisms such as by changing the anatomical localization of B cells and their expression of glycosylation enzymes^35^. In humans, the role of Tbet in B cells is still unclear and not associated with lack of a particular antibody subclass, although inherited T-bet deficiency was associated with skewed class-switching, including reduced IgG2 levels, and lack of a distinct CD11chi, FCRL5hi, CD21lo B-cell subset^50^ Notably, The DDA/TDB/3M-052 liposomes induced high frequencies of T-bet+ B cells at the pre-GC B-cell stage in mice, as described in other settings^42^. T-bet+ memory B cells were described as self-renewing and generated multi-lineage effector cells^51^ and IgG2a/c and IgG1 secreting cells were transcriptionally distinct^52^, had differential tissue residency and recirculation properties^7^ and required different survival signals when differentiated to memory B cells^42^. Thus, the temporal T-bet expression induced by DDA/TDB/3M-052 may tune epigenetic programming and long-term B-cell responses.

Since DDA/TDB/3M-052-induced IgG2c levels did not correlate with Th1 responses, we investigated the mechanism behind B-cell activation and IgG2c switching. scRNA-seq analysis revealed that incorporation of 3M-052 in DDA/TDB led to early upregulation of interferon stimulating genes in draining LN B cells, consistent with a recent study showing that alum-3M-052 led to an elevated antiviral type I IFN response in ILCs and NK cells^53^. Transcriptional analysis also demonstrated stimulation of class-switch regulating genes in a cluster of B cells in the DDA/TDB/3M-052 group. Further, DDA/TDB/3M-052 liposomes directly stimulated B220+CD38+GL7+ pre-GC B cells via TLR7 to initiate T-bet expression, thereby circumventing the need for Th1 cell-derived IFN-γ. Thus, although complete T-bet deficiency abrogated IgG2c switching, the lack of T-bet selectively in T cells did not. These observations are consistent with a recent study showing that IgG2c produced in response to influenza infection does not require T-bet expression in T cells^54^, confirming that there are other mechanisms of IgG2c switching than Th1 induction. Concurrently, we confirm that isolated B cells can be directly stimulated with TLR7 agonists in vitro to produce IgG2c in a T-independent manner (Fig. 5) in line with other in vitro studies^5,55^. In vivo, IgG2c switching in response to T-dependent antigens can be promoted by Th1-derived IFN-γ, but possibly also by IFN-γ from B cells^56^. However, examples where IFN-γ was not required for IgG2a responses have also been described, e.g., for. virus-like particles expressing TLR9 ligands that induced normal IgG2a levels in IFN-γ-deficient mice^23^. Overall, several pathways may facilitate IgG2a/c switching, with the main requirement being the ability to promote T-bet-directed programming in B cells^57^.

In summary, cationic liposomes containing the lipidated TLR7 agonist 3M-052 directly stimulated B-cell activation leading to early class-switching and robust IgG2c Ab responses. This process required T-bet expression, but not Th1 cells. Together with the lack of correlation between Th1 responses and IgG2c switching, this implies that the nature of the adjuvant dictates whether IgG2c/a responses are informative on Th1 responses and that IgG2c responses cannot be used as a reliable general indicator of Th1 immunity. The studies thus highlight DDA/TDB/3M-052 liposomes as a unique adjuvant system for directly activating type I immunity in B cells with little induction of Th1/2/17 responses, which could prove useful in settings where strong effector T-cell responses are undesired, such as in vaccines against HIV infection.

## Methods

### Mice

CB6F1 mice were ordered from Harlan Laboratories (The Netherlands). C57BL/6J, B6.129S1-Tlr7tm1Flv/J (TLR7-/-), B6.129S2-Ighmtm1Cgn (µMT), B6.129S6-Tbx21tm1Glm/J (T-bet-/-), Tcrbtm1Mom/J (TCRβ-/-) and B6(Cg)-Rag2tm1.1Cgn/J (RAG2-/-) mice were ordered from Jackson laboratories (USA). 6–14 week old female mice were used in all experiments. Bone marrow chimeras were generated by transferring bone marrow from the indicated strains (50/50) into RAG2-/- mice intravenously and allowing the mice to rest for 2 months prior to immunization. All mice were housed in the animal facilities at Statens Serum Institut, Denmark. Mouse studies were conducted in accordance with the regulations set forth by the National Animal Protection Committee and in accordance with European Community Directive 86/609. The experiments performed have been approved by the governmental Animal Experiments Inspectorate under licenses 2014-15-2934-01065 and 2017-15-0201-01363. Mice were euthanized by CO2 (80%)/O2 (20%), after anaesthetization with Zoletil-mix (Zolazepam, Tiletamin, Xylazin, and Butorphanol). Humane experimental endpoints included weight loss >20% and signs of distress, including hunched posture, lethargy, unthrifty or stained hair coat, dehydration, dyspnea, hypothermia, and neurological signs.

### Blood donors

Peripheral blood from human study participants was collected after written informed consent, according to Boston Children’s Hospital Institutional Review Board-approved protocol (protocol number 307-05-0223). The mean age of the participants was 29.4 years. All blood samples were de-identified prior to transfer to the lab. Blood samples were anti-coagulated with 15 U/ml pyrogen-free heparin sodium (American Pharmaceutical Partners) and processed within 4 h (typically ∼1–2 h). The number of study participants used for each experimental approach is described in the text.

### Antigens and adjuvants

*C. trachomatis* antigen CTH522 (MOMPextVD4) was recombinantly produced as previously described^58^. Cationic liposomes containing dimethyldioctadecylammonium (DDA) bromide + trehalose 6,6’-dibehenate (TDB) (DDA/TDB liposomes–CAF01) and DDA/TDB/3M-052 were produced in house (Statens Serum Institut, Copenhagen, Denmark) as described^24,27^.

### Immunizations

Mice were immunized subcutaneously (s.c.) at the base of the tail with 2 μg recombinant CTH522 antigen (SSI) in a volume of 200 μl TRIS/trehalose buffer (isotonic, pH 7.4) per immunization. Adjuvant doses were as follows: TDB liposomes (dose 250 μg/50 μg (DDA/TDB)) and TDB + 3M-052 liposomes (dose of 250 μg/50 μg (DDA/TDB)) with incorporation of 3M-052 in doses of 0.4 μg (DDA/TDB/3M-052 _L), 2 μg (DDA/TDB/3M-052 _M), or 10 μg (DDA/TDB/3M-052_H). Control mice (antigen alone) received antigen in 200 μl TRIS-buffer.

### Organ preparation

LNs and spleens were filtered through a 70 μm nylon mesh (BD Biosciences). The cells were washed and prepared as previously described^59^ and re-suspended in cell culture medium (RPMI-1640 supplemented with 5 × 10-^5 ^M 2-mercaptoethnaol, 1% pyruvate, 1% HEPES, 1% (v/v) premixed penicillin-streptomycin solution (Invitrogen Life Technologies), 1 mM glutamine, and 10% (v/v) fetal calve serum (FCS). The cells were adjusted to 2 × 10^5^ cells/well (MSD/ cytokine ELISA) or 1–2 × 10^6^ cells/well (Flow cytometry). Single-cell suspensions were created by homogenizing organs through a 100 µm nylon filter (Falcon). Cell suspensions were centrifuged (700 × *g*, 5 min) and washed twice in RPMI-1640. Cell pellets were re-suspended in RPMI-1640 (Gibco Invitrogen) supplemented with 5 × 10^−5^ M 2-mercaptoethanol, 1 mM glutamine, 1% pyruvate, 1% penicillin-streptomycin, 1% HEPES, and 10% FCS (Gibco Invitrogen).

### Murine in vitro naive B-cell stimulation

B cells were isolated using the Easysep^TM^ pan-B-cell isolation kit (Stemcell Technologies). 4 · 10^5^ B cells/100 μl were incubated in cRPMI with 10% FCS and stimulated with DDA/TDB (final concentration of 50 μg/ml DDA and 10 μg/ml TDB) or DDA/TDB/3M-052 (final concentration of 50 μg/ml DDA, 10 μg/ml TDB and 2 μg/ml 3M-052 in the presence of 12 μl/ml Affinipure F(ab’)2 Fragment Goat anti-Mouse IgM, μ Chain Specific (Jackson Immunoresearch). The cells were stimulated for 24 h for analysis of T-bet by flow cytometry (T-bet PE (4B10), Invitrogen) or 5 days for analysis of IgG2c secretion in the supernatant by ELISA.

### Human in vitro naive B-cell stimulation

Peripheral blood mononuclear cells (PBMCs) were isolated from heparinized whole blood by Ficoll density gradient centrifugation. Naive B cells were isolated by negative selection. Non-naive B cells (CD27+B cells, T cells, NK cells, monocytes, dendritic cells, granulocytes, platelets, and erythroid cells) were labeled with a cocktail of biotinylated CD2, CD14, CD16, CD27, CD36, CD43, and CD235a Abs and magnetically labeled with Anti-Biotin MicroBeads for depletion (Naive B Cell Isolation Kit II, human, Miltenyi Biotec, Auburn, CA). To improve purity, the isolated naive B-cell fraction was subsequently labeled with CD19 microbeads (Miltenyi Biotec) for positive selection, resulting in highly pure naive B-cell populations. Isolated B-cell populations were plated in round-bottom 96-well plates (Corning, Tewksbury, MA, USA) in 100 μL of RPMI-1640 media (Invitrogen; Carlsbad, CA, USA) supplemented with 10% FBS (HyClone, VWR), and stimulated with formulations prepared in an additional 100 μL of media to achieve 1:100 final formulation dilution, and cells were incubated at 37 °C in a 5% CO2 incubator. After 24 h, supernatants were assayed for secreted cytokines by multiplexing bead array, using a customized kit HCYTOMAG-60K (EMD Millipore, Billerica, MA, USA) on a FLexmap 3D instrument (Luminex Corp.; Austin, TX, USA). Cells were analyzed for T-bet expression on an LSRFortessa (Becton), using anti-T-bet (v450, clone O4-46, BD).

### T-cell cytokine profiling

Cytokine responses were measured in supernatants from splenocyte cultures stimulated in vitro with CTH522 antigen (2 µg/ml) in cell culture medium for 72 h at 37 °C and 5% CO2^58^. The Mouse V-plex (custom cytokine: IFN-γ, IL17, IL-5, IL-13) assay was performed according to the manufacturer’s instructions (Meso Scale Discovery). Plates were read on the Sector Imager 2400 system (Meso Scale Discovery) and calculation of cytokine concentrations in unknown samples was determined by 4-parameter logistic non-linear regression analysis of the standard curve

### ELISA for antibody responses

Maxisorb Plates (Nunc) were coated with 0.05 μg/well CTH522 or polyclonal goat anti-mouse IgG (Southern-Biotech), diluted 1:1000, overnight at 4 °C. Individual mouse sera were analyzed in duplicate. After blocking, serum was added in PBS with 2% BSA, starting with a 30-fold dilution for antigen-specific IgG or IgG subclasses. For analysis of total IgG2c secreted by in vitro stimulated murine B cells, the supernatant was added in 10-fold dilutions, starting from undiluted sample. HRP-conjugated secondary antibodies, goat anti-mouse IgG (Zymed), IgG2c (Invitrogen), or IgG1 (Southern Biotech), were diluted in PBS with 1% BSA. After 1 h of incubation, antigen-specific Abs were detected using TMB substrate as described by the manufacturer (Kem-En-Tec Diagnostics). ELISA data were plotted as the sum of absorbances as described previously^60^, as a simple method to visualize antibody responses.

### Flow cytometry

One million cells were stained in PBS + 1% FBS. For ICS, cells were stimulated for 1 h in the presence of CTH522 antigen and 1 µg/ml of costimulatory antibodies CD28 (BD Pharmingen, clone: 37.51) and CD49d (BD Pharmingen, clone: 9C10 (MFR4.B)). Brefeldin A was added at a concentration of 200 µg/ml to each sample and was subsequently incubated at 37 °C for 5 h and kept at 4 °C until staining. Cell suspensions were Fc-blocked with anti-CD16/CD32 antibody (BD Pharmingen, clone 2.4G2, 1:100 dil.) for 10 min. at 4 °C. Cocktails of antibodies against the following surface proteins were used: IgG1 PE (A85-1, 1:1000, BD 550083), B220 PerCP-Cy5.5 (RA3-6B2, 1:500, BD 561101), B220 APC (RA3-6B2, 1:500, BD 553092), B220 FITC (RA3-6B2, 1:500, BD 553087), IgD BV786 (11-26 c.2a, 1:300, BD 563618), GL7 eFluor450 (GL7, 1:300, eBioscience 48-5902-82), CD4 BV786 (GK 1.5. 1:600, BD 563331), IL-2 APC-Cy7 (JES6-5H4, 1:100, BD 560547), IFN-γ PE (XMG1.2, 1:200, BD 554412), TNF-APC (MP6-XT22, 1:200, BD 554420), IgM AF647 (polyclonal, F(ab)2), 1:300, Southern Biotech 1022-31), CD4 BV510 (RM4-5), CD3 BV650 (17A2), CD44 AF700 (IM7), CD38 PE-Cy7 (90), GL7 FITC (GL7), CD4 BV785 (GK1.5, 1:400, Biologend 100559), CD8 BV421 (53-6.7, 1:200, Biolegend 100738), IL-2 APC (JES6-5H4, 1:200, eBioscience 17-7021-82), IFN-γ PE-Cy7 (XMG1.2, 1:200, eBioscience 25-7311-82), TNF-α PE (MP6-XT22, 1:200, eBioscience 12-7321-82), IL-17 PerCP-Cy5.5 (eBio17B7, 1:200, eBioscience 45-7177-82), CxCR3 PerCP-Cy5.5. (CXCR3-173, 1:200, eBioscience 45-1831-82) (eBioscience), CD44 FITC (IM7, 1:600, eBioscience 11-0441-85), T-bet PE (4B10, 1:200, eBioscience 12-5825-82). A live/dead marker was used to exclude dead cells in the antigen-specific B-cell panel and genital tract ICS panel (Fixable Viability Dye eFluor™ 780 or eFluor506, eBioscience). Antigen-specific B cells were measured by including CTH522 coupled to AF488 as a probe (conjugated by Life technologies at a coupling ratio of 3 moles dye/mole). Cells were analyzed on a BD Fortessa or FACSCanto (BD Biosciences) flow cytometer.

### qPCR

B cells from the draining lymph nodes were magnetically isolated by negative selection (STEMCELL). The purity of the isolated B cells was ≥95 % as determined by flow cytometry. RNA purification from murine B cells for qPCR was carried out with RNeasy Mini Kit (Qiagen) and subsequently further purified and concentrated with RNeasy MinElute R Cleanup Kit (Qiagen). The quantity and purity of the isolated RNA were measured with NanoDrop 2000 Spectrophotometer (Thermo Scientific). cDNA synthesis was carried out with the RT2 First Strand kit. qPCR was performed with the RT2 Profiler PCR Array using the specific Mouse Innate and Adaptive Immune Responses array (Qiagen) and a LightCycler® 96 (Roche). Relative gene expression was normalized to five housekeeping reference genes and differential gene expression was calculated as log2 fold-change in gene expression.

### Single-cell RNA sequencing

For each vaccine group, cells from the LN of three mice were pooled and run on the 10x Chromium (10x Genomics) following a library preparation with Chromium Next GEM Single-cell 3’ Reagent Kit v.3.1 (10x Genomics). Libraries were sequenced on the NovaSeq 6000 with the NovaSeq 6000 SP reagent kit v1.5 (Illumina). BCL files were converted to FASTQ files with bcl2fastq (Illumina) and data were then processed through Cellranger (10x Genomics, v6.1.2) and aligned to the mm10 genome. The raw transcript count matrix generated from alignment was loaded into R (v4.3.0) using the Seurat (v.4.3.0) package^61,62^. A Seurat object was created (min.cells = 3, min.features = 200). Data were filtered (nFeature_RNA > 300 & nFeature_RNA < 3000 & percent.mt < 5), normalized (NormalizeData function from Seurat), and variable features were identified (FindVariableFeatures, selection.method = vst). Doublets were filtered out using DoubletFinder (v2.0.3)^63^. The three samples were integrated (FindIntegrationAnchors, IntegrateData from Seurat), scaled (ScaleData), and dimension-reduced by PCA (RunPCA). The first 13 PCs were used to construct an SNN network and a graph-based clustering approach. Louvain algorithm was applied to identify cell clusters with the resolution set to 0.7. UMAP was constructed (RunUMAP), and differentially expressed markers for each cluster were found (FindAllMarkers, min.pct = 0.25, logfc.threshold=0.25, test.use = wilcox). The three B-cell clusters were subset and re-clustered (normalized, variable features were identified, integrated, scaled and PCA transformed). The first 13 PCs were used to construct the UMAP (resolution = 0.5).

### Statistical analysis

Differences between adjuvanted groups were analyzed by One-way ANOVA, with Dunnett´s multiple comparisons test (using the DDA/TDB group as reference). Comparisons of the two groups employed a two-tailed unpaired t-test. Prism 7 software (GraphPad v7.04) was used for all statistical analyses. For scRNA-seq the Seurat (v.4.3.0) package was used.

### Supplementary information

Supplementary information

## Data Availability

All data supporting the findings of this study are available within the paper and its Supplementary Information. Raw data files are available on request. scRNA-seq data were deposited into the Gene Expression Omnibus database under accession number GSE245073.
