# Author Correction: Co-adjuvanting DDA/TDB liposomes with a TLR7 agonist allows for IgG2a/c class-switching in the absence of Th1 cells

**DOI:** 10.1038/s41541-024-00804-4

**Published:** 2024-01-11

**Authors:** Julie Zimmermann, Simon D. van Haren, Joann Diray-Arce, Ignatius Ryan Adriawan, Katharina Wørzner, Ricki T. Krog, Safia Guleed, Tu Hu, Rasmus Mortensen, Jes Dietrich, Sara M. Ø. Solbak, Ofer Levy, Dennis Christensen, Gabriel K. Pedersen

**Affiliations:** 1 Center for Vaccine Research, Statens Serum Institut, Copenhagen, Denmark; 2 Precision Vaccines Program, Boston Children’s Hospital, Boston, MA 02115 USA; 3 Department of Pediatrics, Harvard Medical School, Boston, MA USA; 4 Department of Drug Design and Pharmacology, University of Copenhagen, Copenhagen, Denmark; 5 Broad Institute of MIT and Harvard, Cambridge, MA USA; 6 Department of Immunology and Microbiology, University of Copenhagen, Copenhagen, Denmark

**Keywords:** Adjuvants, Antibodies, Lymphocyte differentiation

Correction to: *npj Vaccines* 10.1038/s41541-023-00781-0, published online 22 December 2023

In this article, the author’s name Joann Diray-Arce was incorrectly written as Joann Arce. The original article has been corrected.

