# Supplementary material for: Co-adjuvanting DDA/TDB liposomes with a TLR7 agonist allows for IgG2a/c class-switching in the absence of Th1 cells
Source: NPJ Vaccines. 2023 Dec 22;8:189. doi: 10.1038/s41541-023-00781-0 (PMC10746746; doi:10.1038/s41541-023-00781-0)
Supplement: Supplementary file 1 — Supplementary information [file 41541_2023_781_MOESM1_ESM.pdf]

Supplementary information

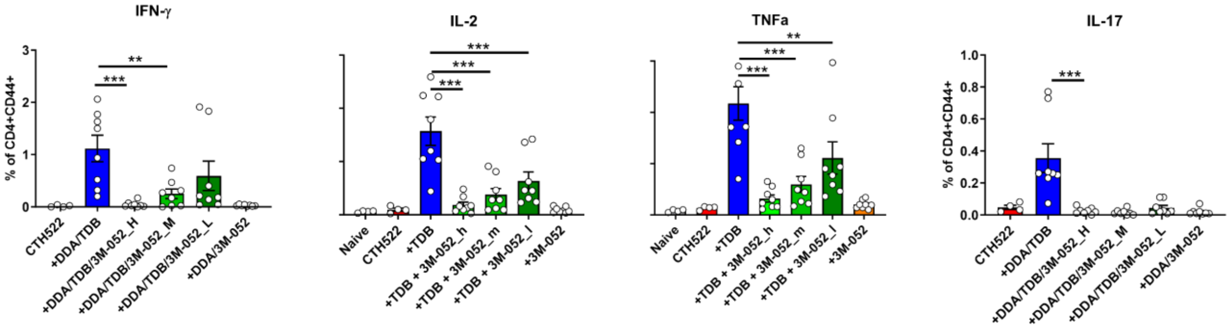

Supplementary Figure 1. Splenic Th1 responses are impaired when 3M-052 is incorporated in DDA/TDB

CB6F1 mice were vaccinated subcutaneously with 2ug of the recombinant protein antigen CTH522 either alone in the presence of the indicated cationic liposomal adjuvant. Adjuvants tested were cationic DDA liposomes containing either TDB (DDA/TDB), 3M-052 (DDA/3M-052) or both (DDA/TDB/3M-052). DDA/TDB/3M-052 was tested with three different doses of 3M-052 (H, M, L -corresponding to a dose of 10ug, 2ug or 0.4ug, respectively). Mice were immunized twice, with three weeks apart, and sacrificed three weeks after the second immunization. Splenic CD4 T cell responses were measured by intracellular staining of the indicated cytokines by flow cytometry. The cells were gated as CD4+CD44+. Groups consisted of 4 (antigen alone) or 8 (antigen +adjuvants) mice. The experiment was performed once. Each point represents individual mice (bars indicate mean+ SEM). Statistically significant differences between groups are indicated by ** and *** (One-way ANOVA with Tukey´s correction for multiple group comparison, using the DDA/TDB group as reference and significance levels of p<0.01 and p<0.001 respectively).

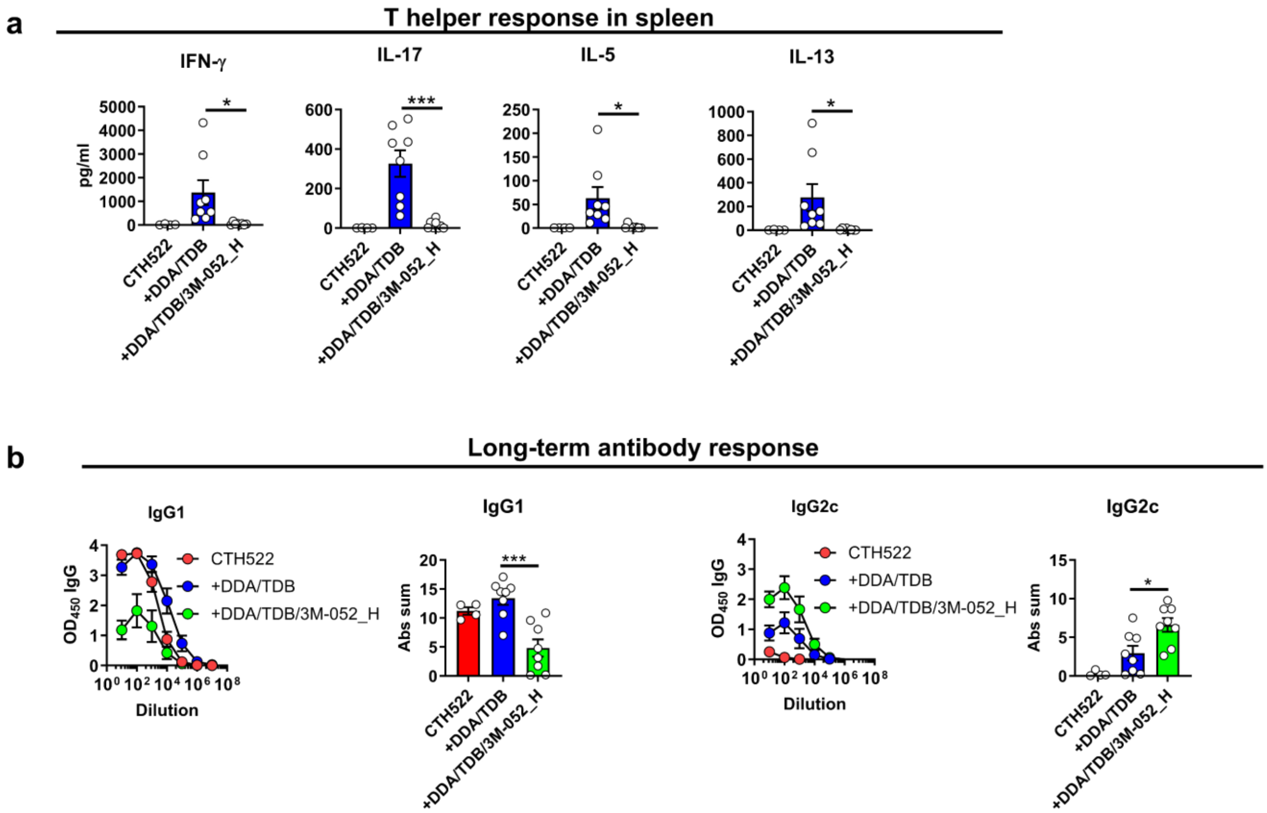

Supplementary Figure 2. Incorporating 3M-052 into DDA/TDB liposomes abrogates Th1/Th17 responses

Mice were vaccinated subcutaneously with 2ug of the recombinant protein antigen CTH522 either alone in the presence of the indicated cationic liposomal adjuvant. Mice were immunized twice and sacrificed three months after the second immunization. **A)** T helper cell responses in the spleen were measured by re-stimulation with antigen and measuring secreted IFN-γ, IL-17, IL-5 and IL-13. **B)** Antibody responses were measured in serum by ELISA and displayed as the ratio between IgG2c and IgG1 responses. The experiment was performed once. Data are depicted as mean+SEM. Statistically significant differences between groups are indicated by * and *** (Two-tailed unpaired t-test, with significance levels of $p<0.05$ and $p<0.001$ respectively).

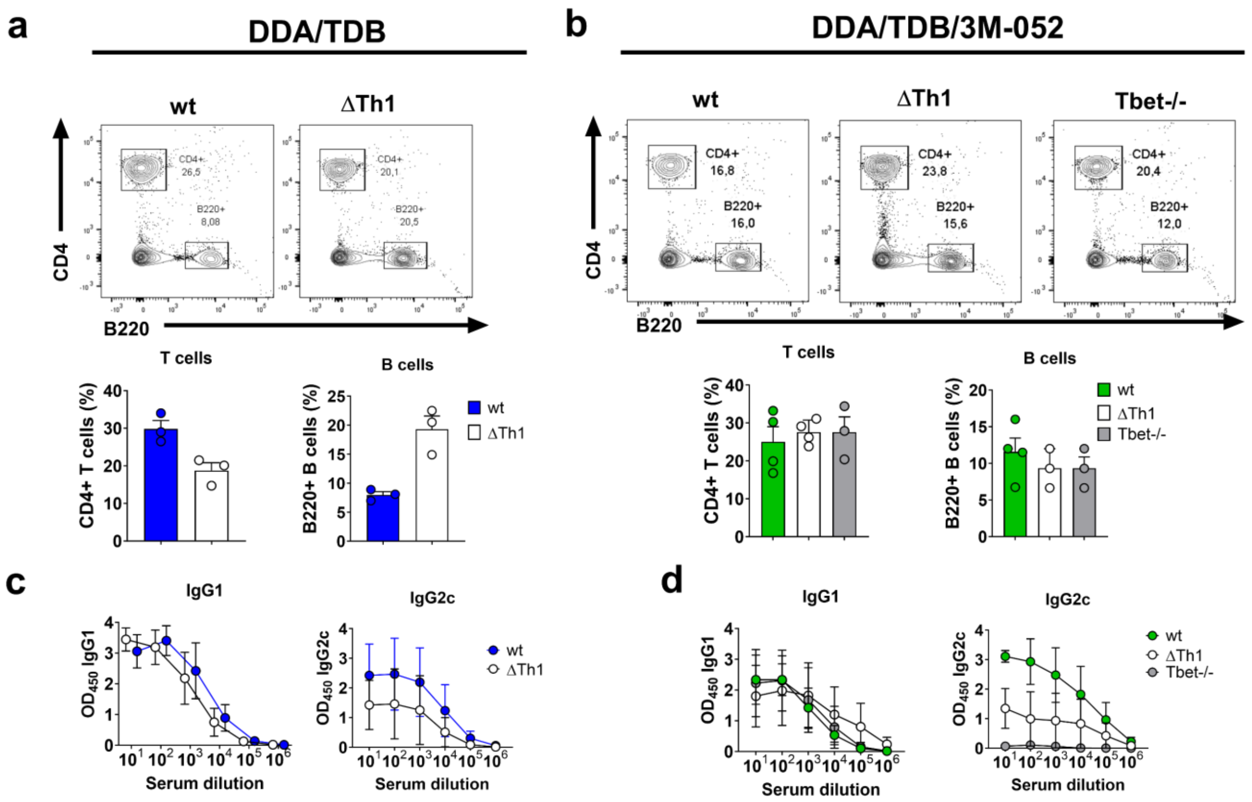

Supplementary Figure 3. IgG2c switching requires T-bet but not Th1 cells

Bone marrow chimeras lacking Th1 cells, or lacking T-bet in both T and B cells, were generated by cell transfer to RAG1-/- mice. The mice were immunized with the CTH522 antigen formulated in cationic DDA liposomes containing either TDB (DDA/TDB) or TDB and 3M-052 (DDA/TDB/3M-052), using the medium dose of 3M-052 (2µg). The mice received three doses each three weeks apart. **A)** Frequencies of CD4+ T cells and B220+ B cells in blood measured one week after the second immunization. **B)** Antigen-specific IgG1 and IgG2c responses measured in serum three weeks after the third immunization with TDB (DDA/TDB) (left panel) or TDB (DDA/TDB/3M-052) (right panel). Groups consisted of 3 - 4 mice and data are depicted as mean+SEM. The experiment was performed once.
